# A Global Perspective of Correlation Between Maternal Copper Levels and Preeclampsia in the 21st Century: A Systematic Review and Meta-Analysis

**DOI:** 10.3389/fpubh.2022.924103

**Published:** 2022-06-27

**Authors:** Zixing Zhong, Qingmei Yang, Tao Sun, Qianqian Wang

**Affiliations:** ^1^Department of Obstetrics, Center for Reproductive Medicine, Zhejiang Provincial People's Hospital (Affiliated People's Hospital, Hangzhou Medical College), Hangzhou, China; ^2^Bengbu Medical College, Bengbu, China; ^3^Department of Reproductive Endocrinology, Center for Reproductive Medicine, Zhejiang Provincial People's Hospital (Affiliated People's Hospital, Hangzhou Medical College), Hangzhou, China; ^4^Department of Obstetrics, Anji Maternity and Child Healthcare Hospital, Huzhou, China

**Keywords:** copper, Cu, trace elements, hypertensive disorder complicating pregnancy, preeclampsia (PE), systematic review, meta-analysis

## Abstract

**Background:**

Preeclampsia (PE) is a common multi-system disorder in pregnancy and a major cause of maternal and perinatal morbidity and mortality globally. Copper is a crucial micronutrient for human health.

**Methods:**

A systematic review was performed according to Preferred Reporting Item for Systematic Reviews and Meta-analysis (PRISMA) guidelines to synthesize the best available evidence regarding the correlation between maternal copper levels and PE from women with different geographical and economic backgrounds.

**Results:**

A total of 34 studies containing 2,471 women with PE and 2,888 healthy pregnant controls across 16 countries were included for research. All studies were systematically reviewed and assessed with the Newcastle-Ottawa Scale (NOS), The Agency of Healthcare for Research and Quality (AHRQ) assessment tools according to the study types. Globally, there was no significant difference in maternal serum copper levels between women with PE and control (Mean difference 5.46, 95% CI −9.63, 20.54). Sub-group analysis from geographical and economic perspectives revealed contrasting results. In conclusion, copper is associated with PE, but the levels of copper leading to increased risk of PE varied across regions and economic development.

**Conclusions:**

The deranged maternal copper levels are correlated with risks of PE, but it presents variously across different geographical and economic contexts.

**Systematic Review Registration:**

https://www.crd.york.ac.uk/prospero/display_record.php?RecordID=306536. Identifier: CRD42022306536.

## Introduction

Preeclampsia (PE) is a subset of hypertensive disorder complicating pregnancy (HDCP) that occurs in around 5% of all pregnancies worldwide (1). It is the leading cause of maternal and perinatal mortality, which has caused particular economic and healthcare burdens in low- and middle-income countries (LMICs) owing to insufficient medical resources.

This maternal disorder often presents in many ways across different organs and systems after 20 weeks of gestation. New-onset hypertension with proteinuria with or without edema is the most common clinical manifestation. However, the etiology of PE is still little known by far. Many candidate factors, such as genetic predisposition, lifestyle factors, and socioeconomic status, can contribute to the pathogenesis of PE (1–3).

Some researchers have proposed that the imbalanced micronutrients in women can increase the risk of PE (3–5). There are discrepancies in the human body status of micronutrients across people from different regions and ethnic backgrounds. Opinions vary to explain the causes. One argument holds that ethnic and genetic background is the main contributor to different serum micronutrient levels, predisposing pregnant women to varying risks of PE (2, 6). However, other academicians believe that the local environment and lifestyle mainly determine the levels of trace elements in inhabitants, which further affects the regional prevalence of PE (3).

Copper is an essential trace element for human health. It functions as a cofactor in enzymes, involved in cell oxidative balance and inflammatory and immune processes. Its deficiency is associated with diabetes and dyslipidemia, while its excessive storage could lead to tumorigenesis (7, 8). In pregnancy, aberrant maternal copper levels may lead to early spontaneous miscarriage, fetal structural anomalies, gestational diabetes, small-for-gestation babies, and preterm birth (9–11). However, its role in the development of PE seems controversial. Some studies reported that maternal Cu-deficiency is associated with an increased risk of copper, while others reported the opposite (5, 11–14). Whether aberrantly high levels of copper or marked low levels are more prone to develop PE is unknown by far. In this article, we searched extensive studies with no limitation of countries and ethnic backgrounds to determine: (1) whether pregnant women from different countries have varied copper levels; (2) whether aberrant serum copper levels are associated with higher maternal risk of PE; (3) how copper levels lead to a higher risk of PE.

## Methods

### Literature Searches

The analysis was performed according to the Preferred Reporting Item for Systematic Reviews and Meta-analysis (PRISMA) statement. The review was registered with PROSPERO (ID: CRD42022306536). Two independent reviewers (ZX-Z and QM-Y) searched six electronic databases (PubMed, Embase, Web of Science, ClinicalTrials.gov, and two Chinese databases, namely, Wanfang and the Chinese National Knowledge Infrastructure, CNKI) from database inception to 26 Jan 2022. The language is limited to English and Chinese. We used a combination of Medical Subject Headings (MeSH) terms and free text words such as “preeclampsia or pre-eclampsia,” “copper or Cu.” We use Chinese words equivalent to the MeSH to search in the Chinese database of Wanfang and CNKI. The search strategy is given in detail in Supplementary 1. We also manually searched the references cited in each included article to complement our initial search.

### Selection Criteria

The criteria for inclusion and exclusion of studies were established prior to the literature search. Studies that fulfilled the following were included: (1) observational studies involving copper and preeclampsia; (2) the exposure of interest should include copper while the outcome of interest should include preeclampsia; (3) the control should be pregnant women instead of non-pregnant women.

A study was excluded if it (1) did not contain serum copper levels, e.g., only described the placental and/or umbilical copper levels; (2) was conducted before the year 2000; (3) the unit of serum copper or the copper levels it gave cannot be converted to mean ± SD.

Study selection was made by two independent investigators (ZX-Z and QM-Y) who carefully reviewed all the articles and determined the inclusion and exclusion criteria by fully discussing them with each other. A third researcher (QQ-W) would resolve any disagreement between the two.

### Quality Assessment and Data Extraction

Quality assessment of each study was based on the Newcastle-Ottawa Scale (NOS, http://www.ohri.ca/programs/clinical_epidemiology/oxford.asp) for case-control and cohort studies, a nine-star rating system to assess the three domains, i.e., selection, comparability, and outcome of a study. A score of 7–9 is considered high quality, 4–6 as fair quality, 0–3 as poor quality. The Agency for Healthcare Research and Quality (AHRQ) checklist (http://www.ncbi.nlm.nih.gov/books/NBK35156/) was used for cross-sectional studies. An item would score 1 point if the study being assessed clearly answers the question, which is marked as a “yes;” the answer of no or not accessible (NA) has no score in the item. A combined score between 8 and 11 indicates good quality, 4–7 fair quality, and 0–3 poor quality. Two individual reviewers (ZX-Z and QM-Y) did the data extraction while disagreements were fully discussed until all three researchers (ZX-Z, QM-Y, and QQ-W) reached a conclusion.

### Sub-group Analysis and Meta-Regression

The influence of various factors, such as the year, location, and type of the study, and the economic level, fasting status, and methods of measurement for copper levels in the study population were explored by performing subgroup analysis and meta-regression.

### Statistical Analysis

The statistical analysis was performed *via* Review Manager 5.4.1 (The Nordic Cochrane Center, Copenhagen, Denmark) and Stata version 15.0 (StataCorp, College Station, TX, USA). The pooled outcomes of serum copper levels were calculated to assess the correlation with PE. All different units of serum copper levels were converted to ug/dl according to the website (http://www.scymed.com/en/smnxtb/tbcbdlb1.htm) with mean ± SD put in the table of the software. The *I*^2^ was used to test the heterogeneity (*I*^2^ ≥ 50% indicates significant heterogeneity), then visualized *via* a forest plot. The random-effect model (REM) was adopted to calculate the combined results if the heterogeneity is considered significant. A sensitivity analysis was performed with the removal of each study once to assess whether any single study could affect the whole outcome. Publication bias was visualized *via* funnel plot with Begg's test and tested with Egger's linear regression.

## Results

### Literature Search

**Figure 1:**
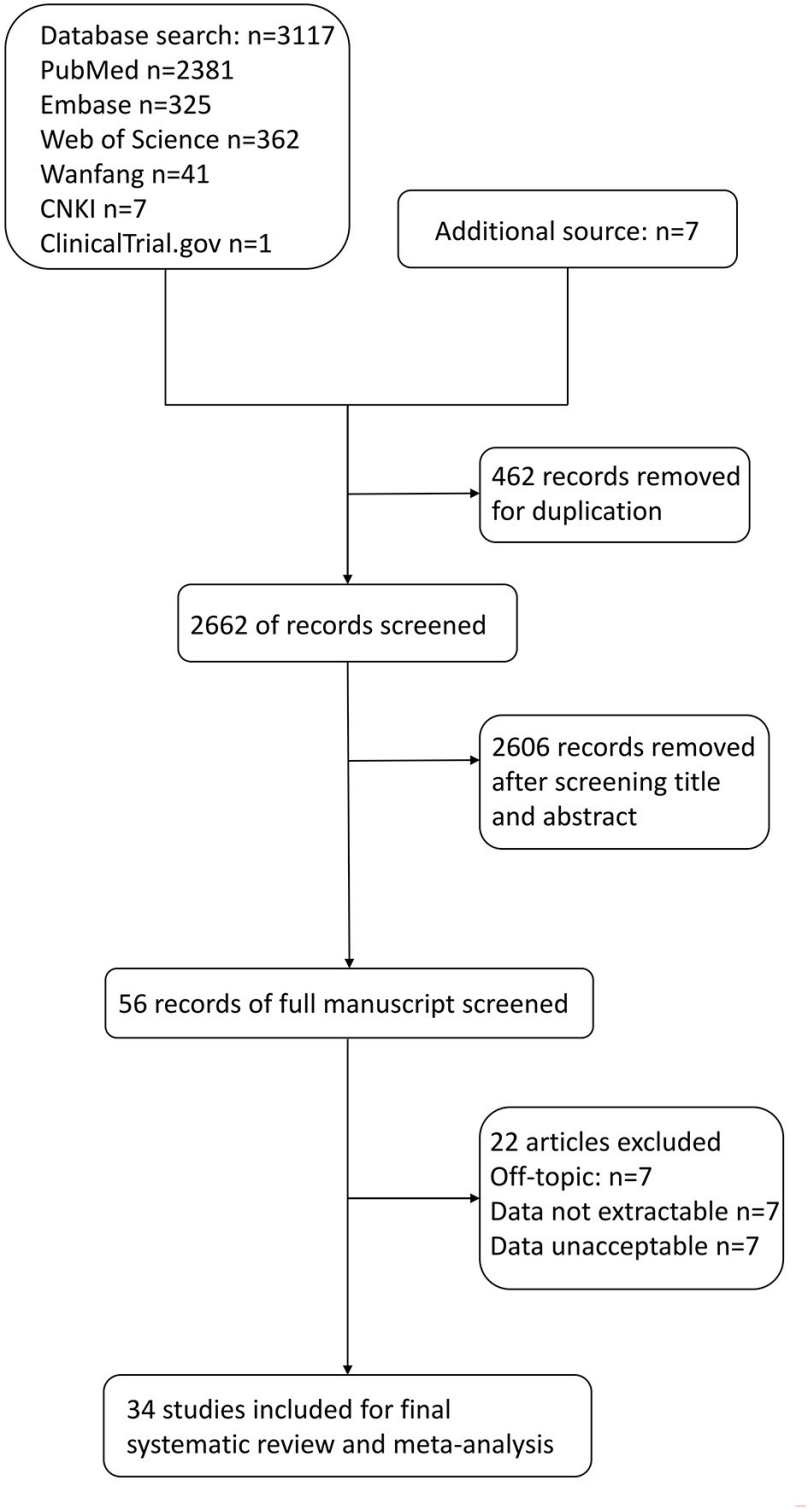
Flow diagram for selection of studies on correlations between maternal copper levels and preeclampsia (PE) in pregnancies.

A total of 3,117 articles were identified through the six electronic databases mentioned above (Figure 1). The other 7 studies were manually added from the reference of some included articles to the study group for further analysis. Of the total 3,124 articles, we excluded 462 duplication reports. A total of 2,606 references were further excluded based on the title and abstract. After the full-text assessment, another 22 articles were excluded, leaving 34 studies in the final group for qualitative and quantitative analysis. Of the 22 articles, seven were excluded for not answering the research question. Two were excluded because the data of PE cannot be extracted separately (15, 16). In comparison, five articles were excluded for inaccessible data. Shaikh et al.'s study was excluded because part of its participants overlapped with an included study (17, 18). The rest of the seven studies were excluded for unacceptable levels of serum copper compared with the normal range in pregnancy updated in an article by Alvarez et al. in 2007 and Zhang et al. in 2013 or not giving units in the article (16, 19–26).

### Characteristics of Included Studies

Thirty-four studies published between 2002 and 2022 were eligible for final review. Twenty-five studies are case-control, six are cross-sectional studies, and three are cohort studies. The detailed characteristics of included studies are presented in Supplementary Table 1. The diagnostic criteria were most commonly referred to as ACOG guidelines in different editions due to the study's years. Some use the local diagnosis of PE which was similar to ACOG's diagnostic criteria (14, 27–31). The number of participants with PE across different studies ranged from 14 to 427, while the number of healthy pregnant control ranged from 23 to 472. Serum copper levels were measured *via* atomic absorption spectrophotometer (AAS) or flame atomic absorption spectrophotometer (FAAS) in 24 studies, while inductively coupled plasma mass spectrometry (ICP-MS) was conducted in four studies (4, 5, 11, 32). The conventional method, “inductively coupled plasma optical emission spectrometer (ICP-OES),” was applied in two studies (33, 34). Bai's study did not clearly state the method of measurement (31).

### Results of the Systematic Review

Thirty-four studies were further categorized as case-control, cross-sectional, and cohort studies, and evaluated *via* Newcastle-Ottawa Score (NOS) and the Agency for Healthcare Research and Quality (AHRQ). After the final evaluation, 19 studies (16 case-control and three cohort studies) were assessed as high quality, while the rest of the 15 studies (nine case-control and six cross-sectional studies) were of fair quality. No study was evaluated as poor quality. The detailed score can be accessible in Supplementary Tables 1, 2, and the publication bias can be seen in Supplementary Figure 1.

### Results of the Meta-Analysis

**Figure 2:**
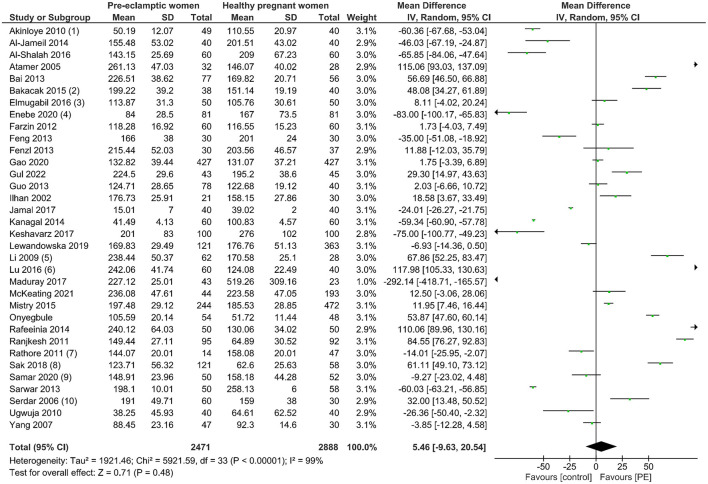
The forest plot of the association between maternal copper levels and PE risk in included studies.

A total of 5,359 participants were enrolled in this analytic study with 2,471 women with PE and 2,888 healthy pregnant controls compared to maternal serum copper levels. From the data we had harmonized from studies across 16 countries, no significant difference in serum copper levels was observed in women with PE compared with normotensive pregnant women (Mean difference 5.46, 95% CI −9.63, 20.54). Details can be seen in Figure 2. Begg's test and Egger's test were applied to assess publication bias, and no significant publication bias was identified (*z* = 1.33, *p* = 0.182; *t* = −0.52, *p* = 0.609; Supplementary Figure 2). Sensitivity analysis showed no single study had influenced the overall effect.

### Results of Meta-Regression

A high level of heterogeneity was observed between studies and in sub-group analysis. Meta-regression was conducted to identify possible causes. The year of the study, study type, geographical location, national income level, fasting status of women upon blood test, and method of measurement were tested for potential contributors of heterogeneity. However, none of the aforementioned variables was significantly associated with the heterogeneity we detected. The *P*-value was 0.51 for the year of the study, 0.48 for study type, 0.47 for the location of the study, 0.87 for economic status, 0.92 for fasting status, and 0.70 for variances in measurement. Detailed results can be seen in Supplementary Figure 3.

### Results of Sub-Group Analysis

#### Sub-Group Analysis From a Geographical Perspective

From a geographical perspective, four studies were from South Asia (two from India, one from Bangladesh, and one from Pakistan, respectively) with 164 participants and 245 controls. The pooled result showed significantly lower levels of copper with a mean difference of −30.45 (95% CI −52.59, −8.31). Twelve studies, including 1,353 participants were originally conducted in the Middle East. A higher level of serum copper was observed in women with PE (mean difference 26.46, 95% CI −2.26, 55.18), the trend would be more prominent after the exclusion of the research from Saudi Arabia, the only regional high-income country in this sub-group (mean difference 32.95, 95% CI 3.57, 62.33). Six studies from Africa (599 participants, 317 with PE vs. 282 controls) also failed to demonstrate a statistically significant difference in copper levels in pregnant women (mean difference −0.82, 95% CI −5.04, 3.41). However, during the sensitivity test, the pooled result can be significantly different (mean difference −46.23, 95% CI −51.95, −40.52) after removing Onyebule et al.'s study. Only two studies purely came from Europe (551 participants all from East Europe), one from Australia (237 participants), and an additional international study from women across Australia, New Zealand, and the UK (4). Detailed results can be seen in Supplementary Figures 4–6.

#### Single Country Sub-group Analysis (China vs. Turkey vs. Iran)

China, Turkey, and Iran ranked top three for most single-country studies. Eight studies focused on Chinese women, six on Turkish, and four on Iran (Supplementary Figure 2). The Chinese and Iranian studies shared a trend of higher levels of serum copper in women with PE (mean difference 24.72 vs. 30.90, 95% CI −3.58, 53.03 vs. −30.79, 92.58, respectively). In Turkey, the copper levels were more pronounced in women with PE than in healthy normotensive mothers-to-be. The mean difference of the national pooled result was 49.95 (95% CI 27.53, 72.37). The forest plot of each of the three countries is shown in Supplementary Figure 7.

#### Sub-group Analysis From an Economic Perspective

**Figure 3:**
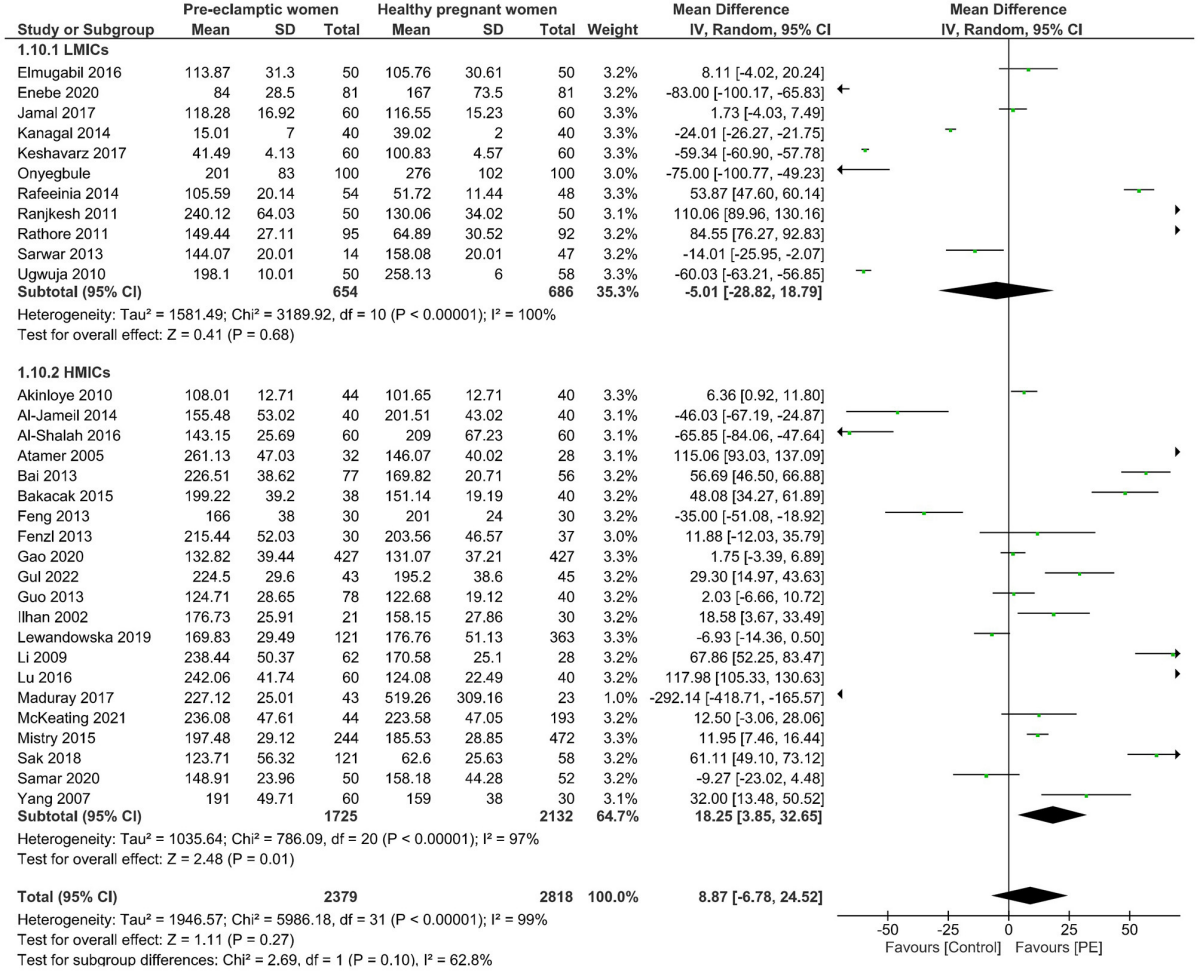
Forest plot of the sub-group analysis. Serum copper levels in women with PE vs. healthy pregnant women from low and lower-middle economies (LMICs) and upper-middle and high-income economies (HMICs).

We then further sub-grouped all the studies according to economic development. The income levels of each country obtained from the World Bank Country classification determined how they were allocated to different sub-groups (35). The sub-groups are low-income economies (36), lower-middle-income economies (Bangladesh, India, Iran, Nigeria, Pakistan), upper-middle-income economies (China, Iraq, South Africa, Turkey), and high-income economies (Australia, Croatia, New Zealand, Poland, Saudi Arabia, UK) (35). No significant difference was observed in copper levels between preeclamptic and non-preeclamptic pregnancies in low-and-lower-middle income economies. However, higher maternal copper levels (mean difference of 17.30, 95% CI 3.60, 30.99) were revealed in women with PE in high-and-upper-middle income economies. The detailed data from the forest plot can be seen in Figure 3 and the funnel plot of this sub-group analysis can be seen in Supplementary Figure 8.

## Discussion

This systematic review and meta-analysis, including 5,359 pregnant women, has shown a correlation between maternal serum copper levels and the risk of PE. However, this is not a unilaterally positive or negative association, and it contradicts all the existing meta-analyses available.

By far, this study has been the most up-to-date systematic review and meta-analysis and has included the largest population and almost all relative studies worldwide since 2000. We consider that the environment, food choices, lifestyle, and the techniques for measuring copper levels are way different compared to that of the 20th century, and this is why we only focus on research in the 21st century. We are the first to combine economic factors and geographic differences concurrently in analyzing the relationship between trace elements and risks of maternal complications during pregnancy. We considered lifestyle factors, socioeconomic status, geographical locations, and ethnicity as contributing factors for PE, and these are supported by current evidence (1, 3, 37). The sub-group analysis was performed accordingly. The results suggested multilateral correlations, which should be interpreted cautiously. In general, a higher level of copper is associated with PE. Women with higher levels of copper from China, Iran, Turkey, the Middle East, and other higher-income level regions are more susceptible to developing PE. In contrast, South Asian and African women seem to experience an opposite trend. This might be explained by a U-shape effect, which suggests that either abnormally low or high levels of copper can lead to a higher risk of PE. Another cause could be that low copper levels indicated the holistic deficiency of trace elements in women in these regions. The imbalanced micronutrient environment makes women susceptible to PE.

Trace elements have long been an essential part of PE research. Calcium has been the hottest topic in preeclamptic research since the 1980 s (38, 39). However, copper has not been fully elucidated for its role in the pathogenesis of PE, despite being the most active antioxidant among biological ions in the human body. It has been found to take an active part in the oxidative stress process in the form of enzymes (40). The altered oxidative stress was observed in the early stage of PE, which results in dysfunctional re-modeling of the uterine spiral arteries, predisposing women to develop preeclamptic symptoms after 20 wks. The studies presenting lower serum copper levels held that a deficiency of copper and its corresponding antioxidant effects in the initial phase of placental formation and formation predispose women to PE later on (5). However, this is not contradicted by some other study findings of higher levels of copper upon the diagnosis of PE or at delivery as copper can be either antioxidant or oxidant (41).

There are two meta-analysis studies in 2016 and 2017 that evaluated the correlation between serum copper level and PE during pregnancy (42, 43). Both showed a higher level of copper associated with an increased risk of PE. However, they included fewer studies, with Song et al. focusing on Asia's population while the other study (42) drew evidence from only 12 articles. Both have missed some articles even in their focus, making the conclusion limitedly valid. In addition, six studies from Turkey were included in the Asian population in Song's meta-analysis. However, the data we analyzed found that Turkey's data are similar to the data extracted from the Middle East. This is consistent with the geographical feature of Turkey, as it is more closely related to Iran, Iraq, and Saudi Arabia than countries such as China, India, and Bangladesh. Furthermore, both the two studies only conduct meta-analyses without systematic review. By contrast, we included more studies with a broader population to avoid selection and publication bias to the minimum level. The extensive work on the systematic review for each article would further consolidate the evidence in our study. The conclusion or the evidence picked up from the sub-group analysis can be of essential importance for researchers with an interest in studying the relationship between copper levels and PE.

However, there are limitations to our study. The between-study heterogeneity is significant even after subgroup analysis and sensitivity analysis. A range of factors could contribute to it. Firstly, the definition of PE varies across studies. Most studies follow the ACOG definition but in different editions owing to the study's year, while others follow the local textbooks (14, 27–30, 32). Some studies have not disclosed how they diagnose PE (18, 44). Secondly, not all studies have clearly stated the timing of blood sampling in women diagnosed with PE. The timings are different in those studies that articulate how and when they did the venipuncture. For example, Lewandowska et al. conducted early-trimester serum copper levels and followed up with late-stage maternal complications, while others performed blood sampling upon the diagnosis or at delivery. Thirdly, we group the nations according to the up-to-date data from World Bank, but not the data of the year of the study, which might cause a minor derange to the overall result from an economic perspective. Fourthly, the unit and method for measuring serum copper levels are different. Various units are used in the measurement, such as ug/dL, mg/L, and umol/L. The blood sample of copper can best reflect the body status of its levels, but the different techniques for measurement can vary across studies, leading to differences in the results of copper levels in women. In some studies, PE was further classified as mild and severe type, or sub-grouped as gestational hypertension, Hemolysis, Elevated Liver enzymes, Low Platelets (HELLP) syndrome, and eclampsia. The results need to be pooled for comparative analysis, which further adds to the heterogeneity of this study.

## Conclusion

In summary, our study demonstrates that maternal serum copper levels and the risk of PE are not simply positively correlated but in complex associations among women from different ethnic and economic backgrounds. Hence, large cohort studies or meta-analyses with individual patient data in the future would identify the role of copper in the pathogenesis of PE.

## Data Availability Statement

The datasets presented in this study can be found in online repositories. The names of the repository/repositories and accession number(s) can be found in the article/Supplementary Material.

## Author Contributions

ZZ and QW: conceptualization, investigation, and resources. ZZ and QY: methodology and formal analysis. ZZ, QY, and TS: software. TS and QW: validation and supervision. QY and TS: data curation. ZZ: writing—original draft preparation and funding acquisition. QW: writing—review and editing and project administration. TS: visualization. All authors have read and agreed to the published version of the manuscript.

## Funding

This research was funded by Zhejiang Provincial Project for Medical and Health Science and Technology, grant number 2022503241.

## Conflict of Interest

The authors declare that the research was conducted in the absence of any commercial or financial relationships that could be construed as a potential conflict of interest.

## Publisher's Note

All claims expressed in this article are solely those of the authors and do not necessarily represent those of their affiliated organizations, or those of the publisher, the editors and the reviewers. Any product that may be evaluated in this article, or claim that may be made by its manufacturer, is not guaranteed or endorsed by the publisher.
