# Supplementary material for: A Global Perspective of Correlation Between Maternal Copper Levels and Preeclampsia in the 21st Century: A Systematic Review and Meta-Analysis
Source: Front Public Health. 2022 Jun 27;10:924103. doi: 10.3389/fpubh.2022.924103 (PMC9271744; doi:10.3389/fpubh.2022.924103)
Supplement: Supplementary file 1 [file Data_Sheet_1.PDF]

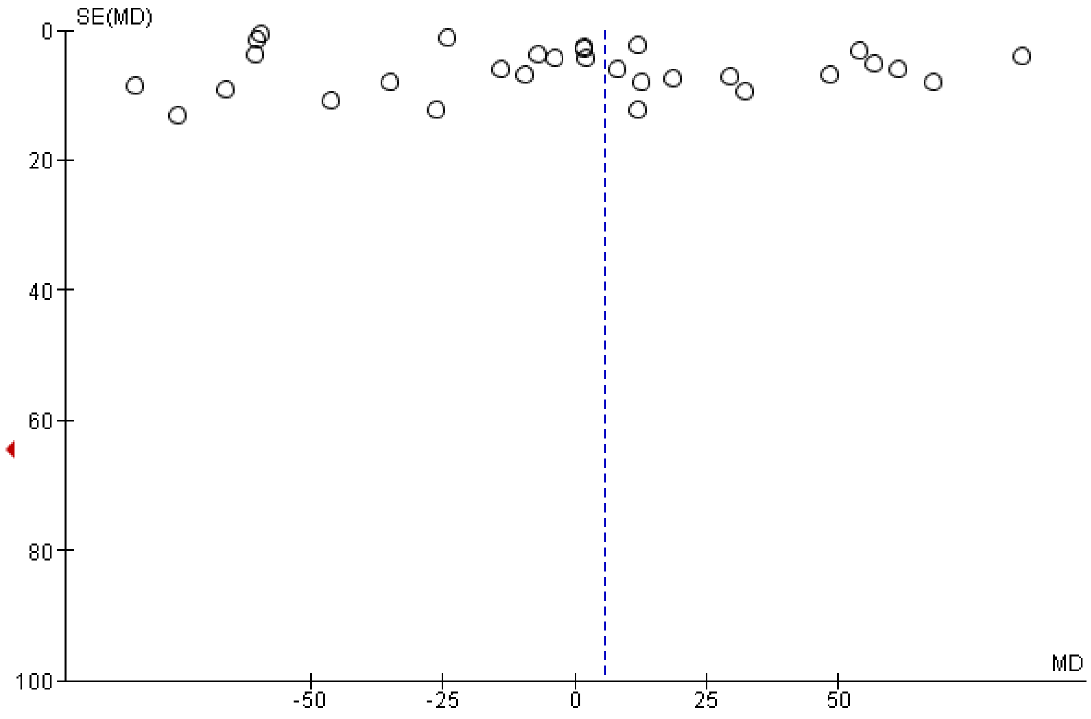

Supplementary Figure 1. Funnel plot of the included studies

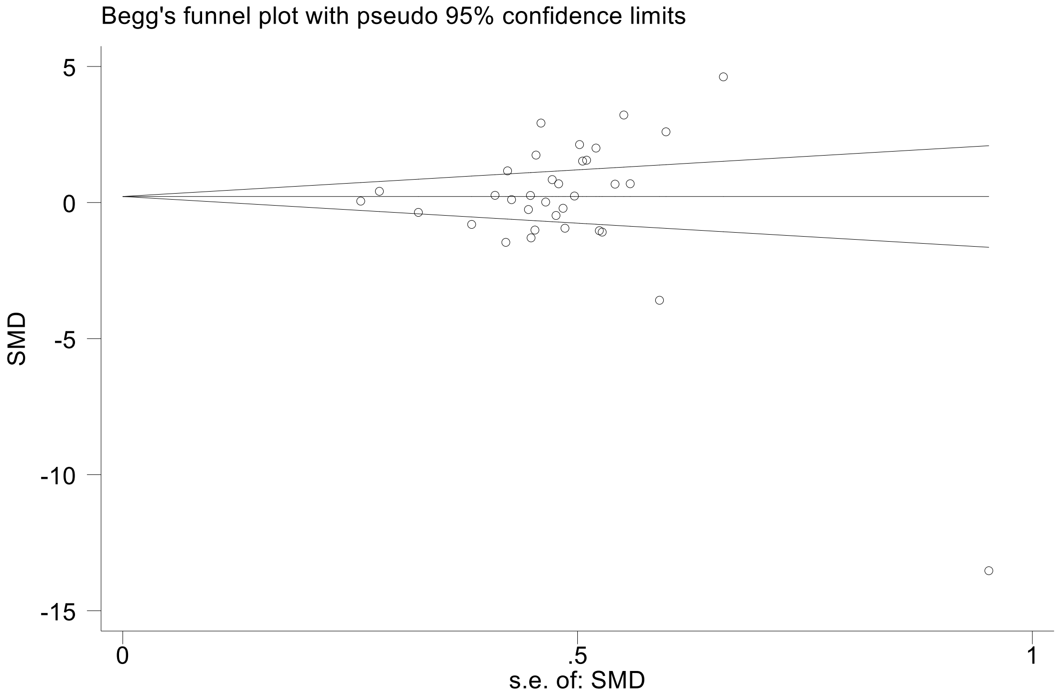

(a)

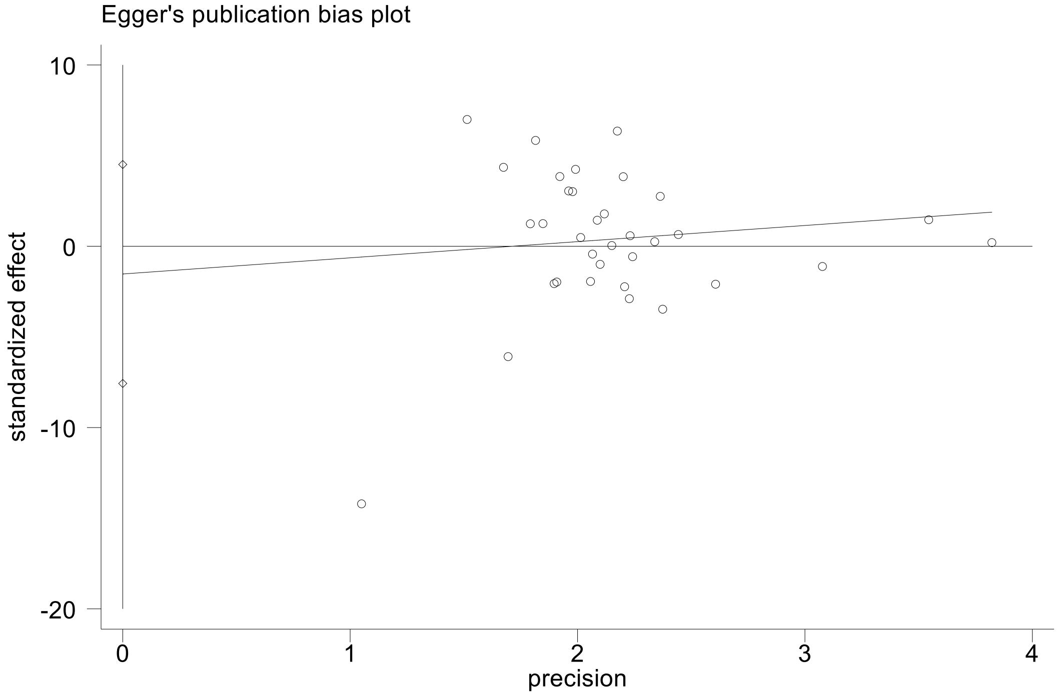

(b)

Supplementary Figure 2. (a). Begg’s test; **(b).** Egger's test for publication bias

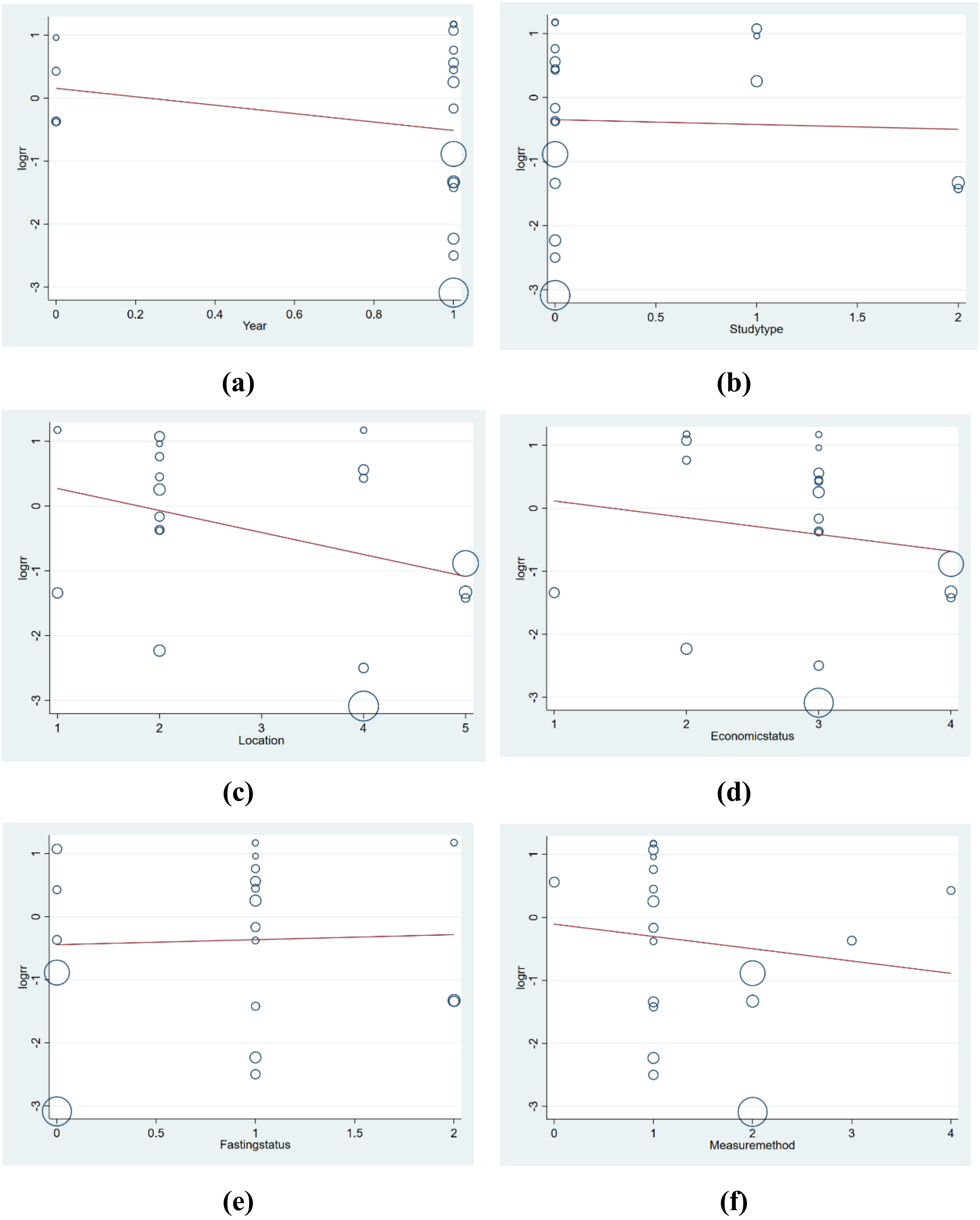

(a) (b) (c) (d) (e) (f)

Supplementary Figure 3. Meta-regressions: the effect size was represented by the logarithm of relative risk (a). the year of the study; (b). study types; (c). the geographical location of the study; (d). the income levels of study; (e). the fasting status of the study; (f). the measuring method.

Study or Subgroup	Pre-eclamptic women Mean	SD	Total	Healthy pregnant women Mean	SD	Total	Weight	Mean Difference IV, Random, 95% CI
Akinloye 2010	50.19	12.07	49	110.55	20.97	40	18.3%	-60.36 [-67.68, -53.04]
Elmugabil 2016	113.87	31.3	50	105.76	30.61	50	18.2%	8.11 [-4.02, 20.24]
Enebe 2020	84	28.5	81	167	73.5	81	18.0%	-83.00 [-100.17, -65.83]
Maduray 2017	227.12	25.01	43	519.26	309.16	23	9.6%	-292.14 [-418.71, -165.57]
Onyegbule	105.59	20.14	54	51.72	11.44	48	18.3%	53.87 [47.60, 60.14]
Ugwuja 2010	38.25	45.93	40	64.61	62.52	40	17.7%	-26.36 [-50.40, -2.32]
**Total (95% CI)**			**317**			**282**	**100.0%**	**-47.34 [-104.13, 9.46]**

Heterogeneity: Tau² = 4585.82; Chi² = 660.76, df = 5 (P < 0.00001); I² = 99%
Test for overall effect: Z = 1.63 (P = 0.10)

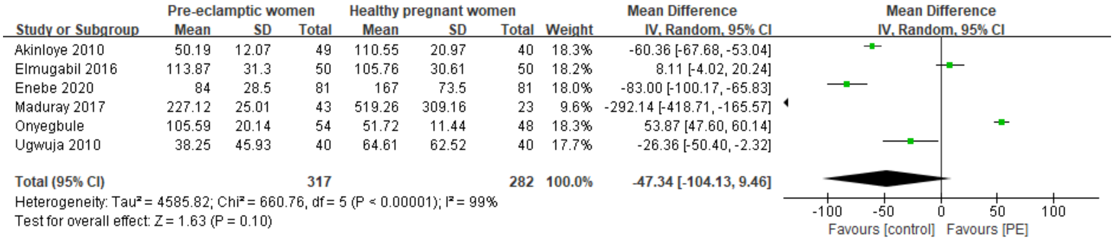

Supplementary Figure 4. Serum copper levels in African pregnant women.

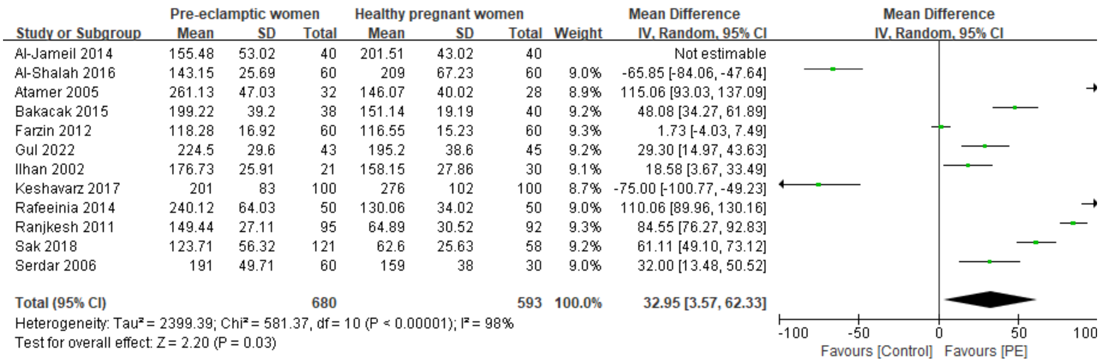

Study or Subgroup	Pre-eclamptic women Mean	SD	Total	Healthy pregnant women Mean	SD	Total	Weight	Mean Difference IV, Random, 95% CI
Al-Jameil 2014	155.48	53.02	40	201.51	43.02	40		Not estimable
Al-Shalah 2016	143.15	25.69	60	209	67.23	60	9.0%	-65.85 [-84.06, -47.64]
Atamer 2005	261.13	47.03	32	146.07	40.02	28	8.9%	115.06 [93.03, 137.09]
Bakacak 2015	199.22	39.2	38	151.14	19.19	40	9.2%	48.08 [34.27, 61.89]
Farzin 2012	118.28	16.92	60	116.55	15.23	60	9.3%	1.73 [-4.03, 7.49]
Gul 2022	224.5	29.6	43	195.2	38.6	45	9.2%	29.30 [14.97, 43.63]
Ilhan 2002	176.73	25.91	21	158.15	27.86	30	9.1%	18.58 [3.67, 33.49]
Keshavarz 2017	201	83	100	276	102	100	8.7%	-75.00 [-100.77, -49.23]
Rafeeinia 2014	240.12	64.03	50	130.06	34.02	50	9.0%	110.06 [89.96, 130.16]
Ranjkesh 2011	149.44	27.11	95	64.89	30.52	92	9.3%	84.55 [76.27, 92.83]
Sak 2018	123.71	56.32	121	62.6	25.63	58	9.2%	61.11 [49.10, 73.12]
Serdar 2006	191	49.71	60	159	38	30	9.0%	32.00 [13.48, 50.52]
**Total (95% CI)**			**680**			**593**	**100.0%**	**32.95 [3.57, 62.33]**

Heterogeneity: Tau² = 2399.39; Chi² = 581.37, df = 10 (P < 0.00001); I² = 98%
Test for overall effect: Z = 2.20 (P = 0.03)

(a)

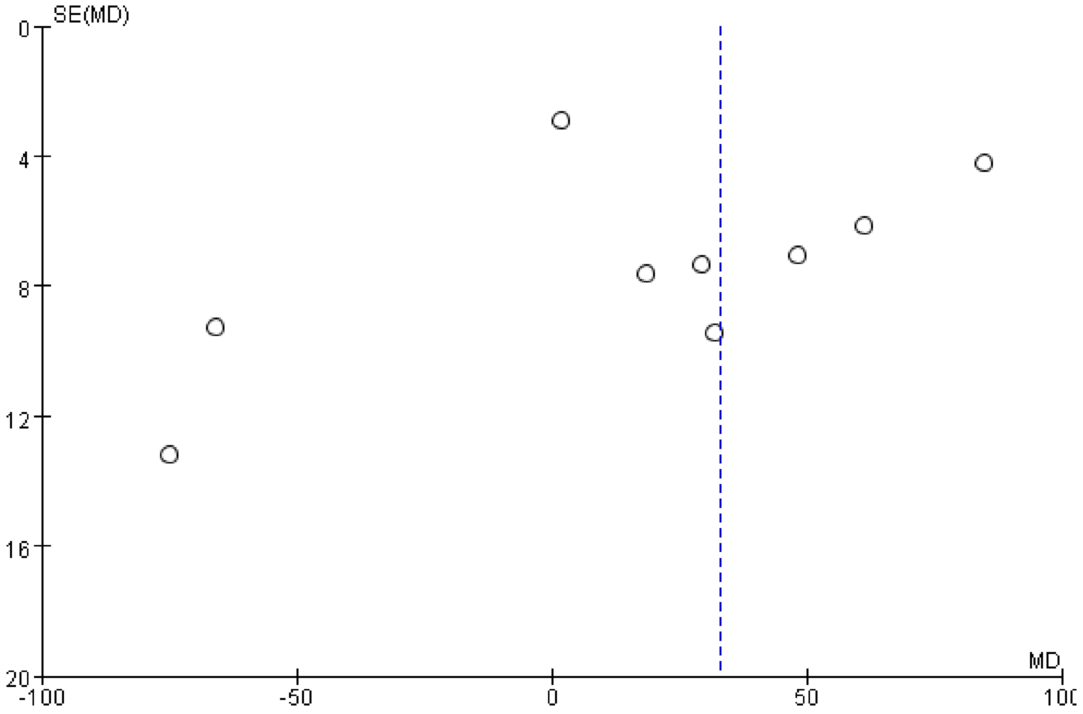

(b)

Supplementary Figure 5. (a). Serum copper levels in Middle-East pregnant women; **(b).** Funnel plot of studies on Middle-East pregnant women.

	Pre-eclamptic women			Healthy pregnant women				Mean Difference
**Study or Subgroup**	**Mean**	**SD**	**Total**	**Mean**	**SD**	**Total**	**Weight**	**IV, Random, 95% CI**
Jamal 2017	15.01	7	40	39.02	2	40	25.5%	-24.01 [-26.27, -21.75]
Kanagal 2014	41.49	4.13	60	100.83	4.57	60	25.5%	-59.34 [-60.90, -57.78]
Rathore 2011	144.07	20.01	14	158.08	20.01	47	23.6%	-14.01 [-25.95, -2.07]
Sarwar 2013	198.1	10.01	50	258.13	6	58	25.4%	-60.03 [-63.21, -56.85]
**Total (95% CI)**			**164**			**205**	**100.0%**	**-39.81 [-60.96, -18.66]**

Heterogeneity: $Tau^2 = 455.82$; $Chi^2 = 719.52$, df = 3 (P < 0.00001); $I^2 = 100\%$

Test for overall effect: Z = 3.69 (P = 0.0002)

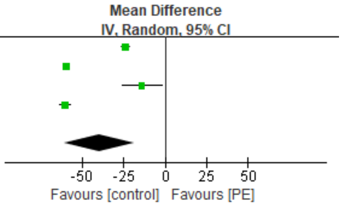

Supplementary Figure 6. Serum copper levels in preeclamptic Vs healthy pregnant women from South Asian

Study or Subgroup	Pre-eclamptic women Mean	SD	Total	Healthy pregnant women Mean	SD	Total	Weight	Mean Difference IV, Random, 95% CI
Bai 2013	226.51	38.62	77	169.82	20.71	56	12.6%	56.69 [46.50, 66.88]
Feng 2013	166	38	30	201	24	30	12.3%	-35.00 [-51.08, -18.92]
Gao 2020	132.82	39.44	427	131.07	37.21	427	12.7%	1.75 [-3.39, 6.89]
Guo 2013	124.71	28.65	78	122.68	19.12	40	12.6%	2.03 [-6.66, 10.72]
Li 2009	238.44	50.37	62	170.58	25.1	28	12.3%	67.86 [52.25, 83.47]
Lu 2016	242.06	41.74	60	124.08	22.49	40	12.5%	117.98 [105.33, 130.63]
Samar 2020	148.91	23.96	50	158.18	44.28	52	12.4%	-9.27 [-23.02, 4.48]
Yang 2007	88.45	23.16	47	92.3	14.6	30	12.6%	-3.85 [-12.28, 4.58]
**Total (95% CI)**			**831**			**703**	**100.0%**	**24.72 [-3.58, 53.03]**

Heterogeneity: Tau² = 1631.74; Chi² = 465.13, df = 7 (P < 0.00001); I² = 98%
Test for overall effect: Z = 1.71 (P = 0.09)

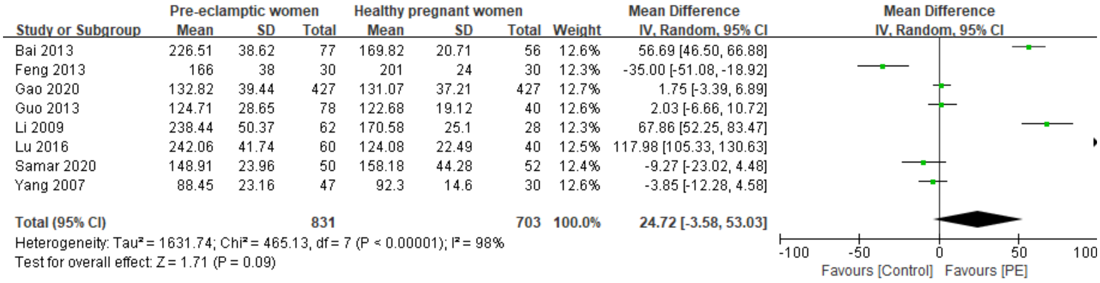

(a)

Study or Subgroup	Pre-eclamptic women Mean	SD	Total	Healthy pregnant women Mean	SD	Total	Weight	Mean Difference IV, Random, 95% CI
Farzin 2012	118.28	16.92	60	116.55	15.23	60	25.4%	1.73 [-4.03, 7.49]
Keshavarz 2017	201	83	100	276	102	100	24.4%	-75.00 [-100.77, -49.23]
Rafeeinia 2014	240.12	64.03	50	130.06	34.02	50	24.8%	110.06 [89.96, 130.16]
Ranjkesh 2011	149.44	27.11	95	64.89	30.52	92	25.4%	84.55 [76.27, 92.83]
**Total (95% CI)**			**305**			**302**	**100.0%**	**30.90 [-30.79, 92.58]**

Heterogeneity: Tau² = 3886.75; Chi² = 383.82, df = 3 (P < 0.00001); I² = 99%
Test for overall effect: Z = 0.98 (P = 0.33)

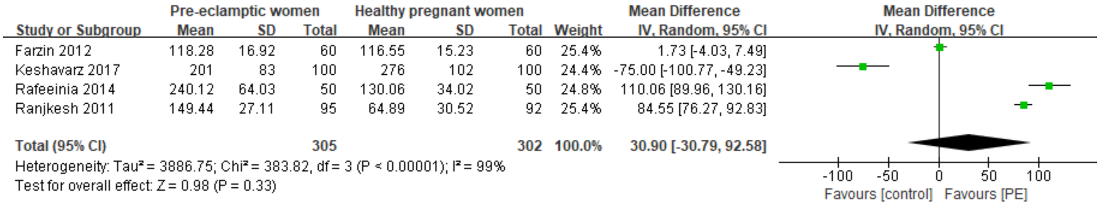

(b)

Study or Subgroup	Pre-eclamptic women Mean	SD	Total	Healthy pregnant women Mean	SD	Total	Weight	Mean Difference IV, Random, 95% CI
Atamer 2005	261.13	47.03	32	146.07	40.02	28	15.5%	115.06 [93.03, 137.09]
Bakacak 2015	199.22	39.2	38	151.14	19.19	40	17.1%	48.08 [34.27, 61.89]
Gul 2022	224.5	29.6	43	195.2	38.6	45	17.0%	29.30 [14.97, 43.63]
Ilhan 2002	176.73	25.91	21	158.15	27.86	30	16.9%	18.58 [3.67, 33.49]
Sak 2018	123.71	56.32	121	62.6	25.63	58	17.3%	61.11 [49.10, 73.12]
Serdar 2006	191	49.71	60	159	38	30	16.2%	32.00 [13.48, 50.52]
**Total (95% CI)**			**315**			**231**	**100.0%**	**49.95 [27.53, 72.37]**

Heterogeneity: Tau² = 716.98; Chi² = 64.31, df = 5 (P < 0.00001); I² = 92%
Test for overall effect: Z = 4.37 (P < 0.0001)

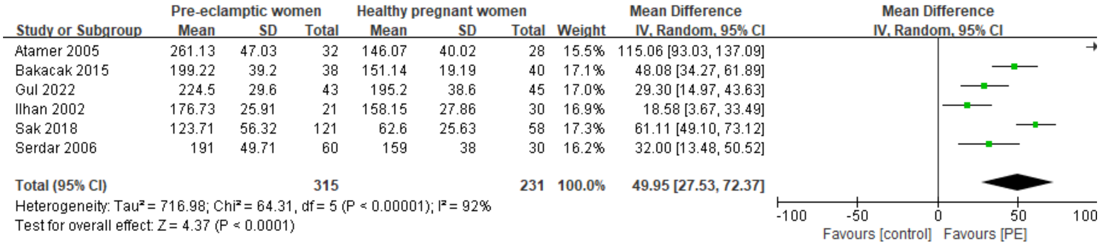

(c)

Supplementary Figure 7. (a). Serum copper levels in pregnant women from China; **(b).** Serum copper levels in pregnant women from Iran; **(c).** Serum copper levels in pregnant women from Turkey.

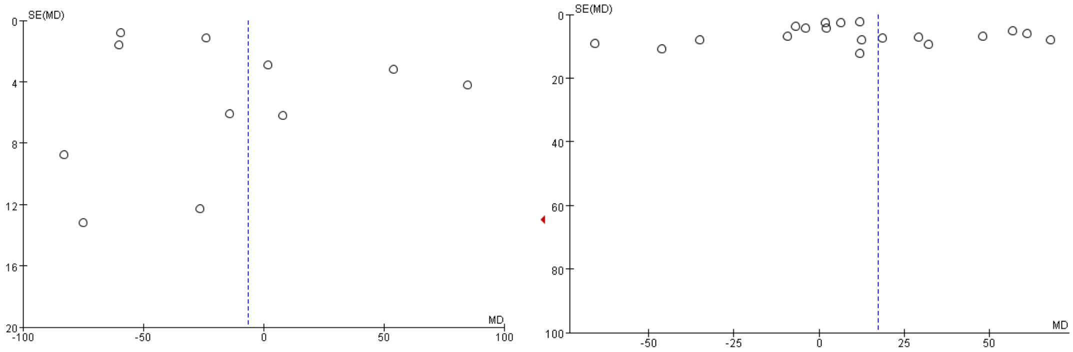

(a) **(b)**

Supplementary Figure 8. (a). Funnel plot of studies on Low-and-Lower-middle income economies; **(b).** Funnel plot of studies on Upper-middle and High-income economies.
