# Supplementary material for: A Global Perspective of Correlation Between Maternal Copper Levels and Preeclampsia in the 21st Century: A Systematic Review and Meta-Analysis
Source: Front Public Health. 2022 Jun 27;10:924103. doi: 10.3389/fpubh.2022.924103 (PMC9271744; doi:10.3389/fpubh.2022.924103)
Supplement: Supplementary file 2 [file Table_1.docx]

**Supplementary Table 1.** Basic characteristics of included studies

| Included studies  (Author + year) | | Country | Study type | Age of PE | | Age of control | | Copper in PE  (Mean±SD) | Copper in control  (Mean±SD) | Data unit | Definition of PE | Measure method |
| --- | --- | --- | --- | --- | --- | --- | --- | --- | --- | --- | --- | --- |
|  |  |  |  | N | (Mean±SD) | N | (Mean±SD) |  |  |  |  |  |
| Akinloye 2010(1) | South Africa | | CS | 49 | NG | 40 | NG | 7.9±1.9 | 17.4±3.3 | umol/L | BP > 140/90 mmHg, proteinuria, platelet aggregation, edema | FAAS |
| Al-Jameil 2014(2) | Saudi Arabia | | CC | 40 | 31.55±6.14 | 40 | 31.20±5.84 | 1.554±0.53 | 2.014±0.43 | mg/L | BP > 140/90 mmHg after 20wks, proteinuria (0.3g/24hrs or 1+ or 100mg/dL by dipstick), platelet aggregation, edema | ICP-OES |
| Al-Shalah 2015(3) | Iraq | | CC | 60 | 27.72±0.66* | 60 | 26.85±0.53* | 143.153±3.316* | 209.657±8.679* | ug/dL | Proteinuria arising de novo after 20^th^ wk | FGAAS |
| Atamer 2005(4) | Turkey | | CS | 32 | 27.00±3.89 | 28 | 25.85±3.36 | 2.612±0.472 | 1.458±0.396 | mg/L | ISSHP(5) | AAS |
| Bai  2013(6) | China | | CC | 41+36 | 28.3±7.6 | 56 | 27.6±7.2 | 35.65±6.08 | 26.73±3.26 | umol/L | Textbook(7) | Not mention |
| Bakacak 2015(8) | Turkey | | CC | 38 | 29.2±3.56 | 40 | 28.7±3.82 | 199.5  (281.86-114.4)** | 152.45  (187.32-104.56) | ug/dL | ACOG(9) | FAAS |
| Elmugabil 2016(10) | Sudan | | CC | 50 | 28.6±6.4 | 50 | 28.6±6.6 | 111.6  (94.3-135.3)*** | 103.6  (86.6-126.7)*** | ug/dL | ACOG(11) | AAS |
| Enebe  2020(12) | Nigeria | | CS | 81 | 29.53±5.38 | 81 | 29.31±5.22 | 0.844±0.29 | 1.670±0.74 | mg/L | ISSHP(13) | AAS |
| Farzin  2012(14) | Iran | | CC | 60 | 27.43±3.91 | 60 | 26.66±3.72 | 118.28±16.92 | 116.55±15.23 | ug/dL | BP > 140/90 mmHg, proteinuria, platelet aggregation | FAAS |
| Feng  2013(15) | China | | CC | 30 | 28.63±6.38 | 30 | 28.73±5.28 | 1.66±0.38 | 2.01±0.24 | ug/mL | Textbook(7) | AAS |
| Fenzl  2013(16) | Croatia | | PC | 30 | 31.2 | 37 | 30.8 | 33.91±8.19 | 32.04±7.33 | umol/L | ACOG(17) | FAAS |
| Gao  2020(18) | China | | CC | 427 |  | 427 |  | 1.31  (1.07-1.60)*** | 1.31  (1.06-1.56)*** | mg/L | ISSHP(13) | ICP-MS |
| Gul  2022(19) | Turkey | | CC | 43 | 30.2±6.8 | 45 | 27.2±5.3 | 224.5±29.6 | 195.2±38.6 | ug/dL | ACOG(20) | Fully automatic photometric method |
| Guo  2013(21) | China | | CC | 26+20 | NG | 40 | NG | 19.39±5.51  18.91±4.02 | 19.31±3.01 | umol/L | Textbook(7) | FAAS |
| Illhan  2002(22) | Turkey | | CC | 21 | NG | 30 | NG | 176.73±25.91 | 158.15±27.86 | ug/dL | Edema, proteinuria (≥2+) and hypertension (≥2 140/95 mmHg) | AAS |
| Jamal  2017(23) | Pakistan | | CS | 40 | 25.76±0.73 | 40 | 25.46±0.85 | 0.39±0.02 | 0.15±0.07 | mg/dL | NG | FAAS |
| Kanagal 2014(24) | India | | CC | 60 | 27.45±4.33 | 60 | 25.87±3.11 | 6.53±0.65 | 15.87±0.72 | umol/L | ACOG(25) | AAS |
| Keshavarz 2017(26) | Iran | | CC | 100 | 28.83±5.94 | 100 | 28.36±3.26 | 2.01±0.83 | 2.76±1.02 | mg/L | Textbook(27) | FAAS |
| Lewandowska 2019(28) | Poland | | PC | 121 | 35.1±4.2 | 363 | 35.1±4.0 | 1595.01±255.24 | 1693.39±275.70 | ug/L | BP≥140/90 mmHg with proteinuria (≥300mg/d or ≥0.3g/L or protein/creatinine ratio ≥0.3 or 1+ in the strip test | ICP-MS |
| Li 2009(29) | China | | CC | 36+26 | 27.90±2.51  26.02±3.10 | 28 | 28.32±3.48 | 34.89±6.02  41.19±8.8 | 26.85±3.95 | umol/L | Textbook(7) | Nitro-PAPS |
| Lu 2016(30) | China | | CC | 30+30 | 29.10±2.40  30.00±2.00 | 30 | 29.60±2.00 | 35.00±3.42  41.20±7.50 | 26.70±3.00 | umol/L | Textbook(7) | AAS |
| Maduray 2017(31) | South Africa | | CC | 43 | 25±5 | 23 | 24±5 | 2.27±0.25  (0.42, 8.21)# | 5.19±3.09  (0.91, 58.59)# | mg/L | New onset hypertension (BP≥140/90 mmHg) and proteinuria (≥300mg/24h) | ICP-OES |
| McKeating 2021(32) | Australia | | NC | 44 | 31.55±3.8 | 193 | 32.24±3.91 | 2.296  (3.675-1.587) | 2.214  (3.783-1.222) | mg/L | NG | ICP-MS |
| Mistry  2015(33) | UK / NZ /  Australia | | NCC | 244 | 28 (23, 32)*** | 472 | 29 (23, 32)*** | 1957.4  (1787, 2177.5)*** | 1850.0  (1663.5, 2051.5)*** | ug/L | BP≥140/90 mmHg with proteinuria (≥300mg/d or ≥0.3g/L or protein/creatinine ratio ≥0.3 or 2+ in the strip test | ICP-MS |
| Onyegbule 2016(34) | Nigeria | | CC | 54 | 27±7.02 | 48 | 29±5.35 | 16.62±3.17 | 8.14±1.80 | umol/L | ACOG(9) | AAS |
| Rafeeinia 2014(35) | Iran | | CC | 50 | 26.50±3.90 | 50 | 27.10±4.60 | 2.40±0.64 | 1.30±0.34 | mg/L | BP>130/85 mmHg and proteinuria (≥1) | AAS |
| Ranjkesh 2011(36) | Iran | | CS | 95 | 26.41±4.91 | 92 | 25.22±5.14 | 149.44±27.11 | 64.89±30.52 | ug/dL | Textbook(37) | AAS |
| Rathore 2011(38) | India | | CC | 14 | NG | 47 | NG | 1.58±0.2 | 1.44±0.2 | mg/dL | New onset of hypertension and proteinuria, edema after 20wks | AAS |
| Sak  2020(39) | Turkey | | CS | 71+26 | 35.7±7.28  36.6±7.72 | 58 | 36.6±6.6 | 81.2±11.84  160.2±20.89 | 62.6±25.63 | ug/dL | ACOG(9) | FAAS |
| Samar  2020(40) | China | | CC | 50 | 29.34±4.35 | 52 | 26.44±4.02 | 23.07  (21.12-26.06)*** | 25.23  (20.19-29.33)*** | umol/L | NG | Chemical light |
| Sarwar 2013(41) | Bangladesh | | CC | 50 | 25.46±0.85* | 58 | 25.76±0.73* | 1.98±0.10* | 2.58±0.06* | mg/dL | New onset of hypertension ±edema after 20wks | FAAS |
| Serdar 2006(42) | Turkey | | CC | 30+30 | 25±2.3  26±1.9 | 30 | 27±4.1 | 188±48  194±52 | 159±38 | ug/dL | New onset of hypertension and proteinuria (≥1) | Randox Laboratoryies^+^ |
| Ugwuja 2010(43) | Nigeria | | CC | 40 | 29.45±3.70 | 40 | 27.55±4.23 | 6.02±7.23 | 10.17±9.84 | umol/L | ACOG(25) | AAS |
| Yang  2007(44) | China | | CC | 17+30 | 28.75±2.64 | 30 | 26.83±2.59 | 0.9192±0.1437  0.8648±0.2694 | 0.9229±0.1460 | ug/mL | Textbook(45) | FAAS |

**NB** NC: Nested cohort; NCC: Nested case-control; ICP-MS: Inductively coupled plasma mass spectrometry; ICP-OES: Inductively coupled plasma optical emission spectrometer.

*Mean ± SEM (standard error of mean); **Median range (Maximum-minimum); ***Median (Inter-quartile); #Median±SEM (Minimum, Maximum); ^+^A commercially available procedure for copper measurement.

**Table 2. Quality assessment (AHRQ) of cross-sectional studies**

|  | Akinloye  2010(1) | Atamer  2005(4) | Enebe 2020(12) | Jamal 2017(23) | Ranjkesh 2011(36) | Sak 2020(39) |
| --- | --- | --- | --- | --- | --- | --- |
| Define the source of information | ★ | ★ | ★ | ★ | ★ | ★ |
| List inclusion & exclusion criteria | ★ | ★ | ★ | ★ | ★ | ★ |
| Indicate time period used for identifying patients | ★ | - | ★ | ★ | - | ★ |
| Indicate whether or not subjects were consecutive if not population-based | ★ | - | ★ | - | - | ★ |
| Indicate if evaluators of subjective components of the study were masked to other aspects of the status of the participants | - | - | - | - | - | - |
| Describe any assessments undertaken for quality assurance purposes | ★ | - | ★ | - | ★ | ★ |
| Explain any patient exclusions from the analysis | NA | NA | NA | NA | ★ | NA |
| Describe how confounding was assessed and/or controlled | ★ | ★ | ★ | ★ | ★ | ★ |
| If applicable, explain missing data were handled in the analysis | NA | NA | NA | NA | NA | NA |
| Summarize patient response rates and completeness of data collection | ★ | ★ | ★ | ★ | ★ | ★ |
| Clarify what follow-up was expected and the percentage of patients for which incomplete data or follow-up was obtained | NA | NA | NA | NA | NA | NA |
| Score | 7 | 4 | 7 | 5 | 6 | 7 |

**NB** AHRQ (The Agency for Healthcare Research and Quality) methodology checklist for cross-sectional studies; NA: not applicable

**Table 3.1 Quality assessment (NOS) of case-control studies**

| Included studies | Selection | | | | | | | Comparability | | Exposure | | | | | Score |
| --- | --- | --- | --- | --- | --- | --- | --- | --- | --- | --- | --- | --- | --- | --- | --- |
|  | ① | | ② | | ③ | | ④ | ⑤ | ⑥ | | | ⑦ | | ⑧ |  |
| Al-Jameil 2014(2) | ★ | |  | |  | |  | ★★ | ★ | | | ★ | | ★ | 6 |
| Al-Shalah 2015(3) | ★ | | ★ | | ★ | | ★ | ★★ | ★ | | | ★ | | ★ | 9 |
| Bai 2013(6) | ★ | | ★ | | ★ | | ★ | ★★ | ★ | | | ★ | | ★ | 9 |
| Bakacak 2015(8) | ★ | |  | | ★ | | ★ | ★★ | ★ | | | ★ | | ★ | 8 |
| Elmugabil 2016(10) | ★ | |  | |  | | ★ | ★★ | ★ | | | ★ | | ★ | 7 |
| Farzin 2012(14) | ★ | |  | |  | | ★ | ★★ | ★ | | | ★ | | ★ | 7 |
| Feng 2013(15) | ★ | |  | | ★ | | ★ | ★★ | ★ | | | ★ | | ★ | 8 |
| Gao 2020(18) | ★ | | ★ | | ★ | |  | ★★ | ★ | | | ★ | | ★ | 8 |
| Gul 2022(19) | ★ | |  | |  | | ★ | ★★ | ★ | | | ★ | | ★ | 7 |
| Guo 2013(21) | ★ | |  | |  | |  | ★★ | ★ | | | ★ | | ★ | 6 |
| Illhan 2002(22) | ★ | |  | | ★ | | ★ | ★★ | ★ | | | ★ | | ★ | 8 |
| Kanagal 2014(24) | ★ | |  | |  | |  |  | ★ | | | ★ | | ★ | 4 |
| Keshavarz 2017(26) | ★ | | ★ | |  | |  | ★ | ★ | | | ★ | | ★ | 6 |
| Li 2009(29) | ★ | |  | |  | | ★ | ★★ | ★ | | | ★ | | ★ | 7 |
| Lu 2016(30) | ★ | |  | | ★ | |  | ★★ | ★ | | | ★ | | ★ | 7 |
| Maduray 2017(31) | ★ | | ★ | |  | |  | ★ | ★ | | | ★ | | ★ | 6 |
| Mistry 2015(33) | ★ | | ★ | | ★ | | ★ | ★★ | ★ | | | ★ | | ★ | 9 |
| Onyegbule 2016(34) | ★ | | ★ | |  | |  | ★ | ★ | | | ★ | | ★ | 6 |
| Rafeeinia 2014(35) | ★ | |  | |  | |  | ★ | ★ | | | ★ | | ★ | 5 |
| Rathore 2011(38) | ★ | |  | |  | |  |  | ★ | | | ★ | | ★ | 4 |
| Samar 2020(40) |  | |  | |  | |  | ★★ | ★ | | | ★ | | ★ | 5 |
| Sarwar 2013(41) | ★ | | ★ | | ★ | | ★ | ★ | ★ | | | ★ | | ★ | 8 |
| Serdar 2006(42) | ★ | | ★ | |  | |  | ★★ | ★ | | | ★ | | ★ | 7 |
| Ugwuja 2010(43) | ★ |  | | ★ | |  | | ★★ | | ★ | ★ | | ★ | | 7 |
| Yang 2007(44) | ★ |  | |  | | ★ | | ★★ | | ★ | ★ | | ★ | | 7 |

**NB** NOS for Newcastle-Ottawa Scale

Selection: ① Is the case definition adequate; ② Representative of the cases; ③ Selection of controls; ④ Definition of controls

Comparability: ⑤ Comparability of cases and controls based on the design or analysis

Exposure: ⑥ Ascertainment of exposure; ⑦ Same method of ascertainment for cases and controls; ⑧ Non-response rate

**Table 3.2 Quality assessment (NOS) of cohort studies**

| Included studies | Selection | | | | Comparability | Outcome | | | Score |
| --- | --- | --- | --- | --- | --- | --- | --- | --- | --- |
|  | ① | ② | ③ | ④ | ⑤ | ⑥ | ⑦ | ⑧ |  |
| Fenzl 2013(16) | ★ | ★ | ★ | ★ | ★★ | ★ | ★ | ★ | 9 |
| Lewandowska 2019(28) | ★ | ★ | ★ | ★ | ★★ | ★ | ★ | ★ | 9 |
| McKeating 2021(32) | ★ | ★ | ★ | ★ | ★★ | ★ | ★ | ★ | 9 |

**NB** Selection: ① Representativeness of the exposed cohort; ② Selection of the non-exposed cohort; ③ Ascertainment of exposure; ④ Demonstration that outcome of interest was not present at the start of study;

Comparability: ⑤ Comparability of cohorts based on the design or analysis;

Outcome: ⑥ Assessment of outcome; ⑦ Was follow-up long enough for outcomes to occur; ⑧ Adequacy of follow up of cohorts

**Reference**

1. Akinloye O, Oyewale OJ, Oguntibeju OO. Evaluation of Trace Elements in Pregnant Women with Pre-Eclampsia. *African Journal of Biotechnology* (2010) 9(32):5196-202.

2. Al-Jameil N, Tabassum H, Al-Mayouf H, Aljohar HI, Alenzi ND, Hijazy SM, et al. Analysis of Serum Trace Elements-Copper, Manganese and Zinc in Preeclamptic Pregnant Women by Inductively Coupled Plasma Optical Emission Spectrometry: A Prospective Case Controlled Study in Riyadh, Saudi Arabia. *International journal of clinical and experimental pathology* (2014) 7(5):1900-10. Epub 2014/06/27.

3. Al-Shalah HH, Al-Hilli NM, Hasan MA. The Association of Serum Iron, Zinc, and Copper Levels with Preeclampsia. *Medical Journal of Babylon* (2015) 12(4):1027-36.

4. Atamer Y, Koçyigit Y, Yokus B, Atamer A, Erden AC. Lipid Peroxidation, Antioxidant Defense, Status of Trace Metals and Leptin Levels in Preeclampsia. *European journal of obstetrics, gynecology, and reproductive biology* (2005) 119(1):60-6. Epub 2005/03/01. doi: 10.1016/j.ejogrb.2004.06.033.

5. Davey DA, MacGillivray I. The Classification and Definition of the Hypertensive Disorders of Pregnancy. *Am J Obstet Gynecol* (1988) 158(4):892-8. Epub 1988/04/01. doi: 10.1016/0002-9378(88)90090-7.

6. Bai T. The Diagnostic Value of Trace Elements, Ldh and Ua in Hypertensive Disorders in Pregnancy. *Chinese Journal of Postgraduate of Medicine* (2013) 36(12):49-52.

7. Le J. *Obstetrics and Gynecology*. 7th ed. Beijing, China: People's Medical Publishing House (2008).

8. Bakacak M, Kılınç M, Serin S, Ercan Ö, Köstü B, Avcı F, et al. Changes in Copper, Zinc, and Malondialdehyde Levels and Superoxide Dismutase Activities in Pre-Eclamptic Pregnancies. *Medical science monitor : international medical journal of experimental and clinical research* (2015) 21:2414-20. Epub 2015/08/19. doi: 10.12659/msm.895002.

9. Pregnancy TFoHi. Hypertension in Pregnancy. Report of the American College of Obstetricians and Gynecologists' Task Force on Hypertension in Pregnancy. *Obstet Gynecol* (2013) 122(5):1122-31. Epub 2013/10/24. doi: 10.1097/01.AOG.0000437382.03963.88.

10. Elmugabil A, Hamdan HZ, Elsheikh AE, Rayis DA, Adam I, Gasim GI. Serum Calcium, Magnesium, Zinc and Copper Levels in Sudanese Women with Preeclampsia. *PloS one* (2016) 11(12):e0167495. Epub 2016/12/03. doi: 10.1371/journal.pone.0167495.

11. Acog Technical Bulletin. Hypertension in Pregnancy. Number 219--January 1996 (Replaces No. 91, February 1986). Committee on Technical Bulletins of the American College of Obstetricians and Gynecologists. *Int J Gynaecol Obstet* (1996) 53(2):175-83. Epub 1996/05/01.

12. Enebe JT, Dim CC, Ugwu EO, Enebe NO, Meka IA, Obioha KC, et al. Serum Antioxidant Micronutrient Levels in Pre-Eclamptic Pregnant Women in Enugu, South-East Nigeria: A Comparative Cross-Sectional Analytical Study. *BMC pregnancy and childbirth* (2020) 20(1):392. Epub 2020/07/08. doi: 10.1186/s12884-020-03081-w.

13. Brown MA, Magee LA, Kenny LC, Karumanchi SA, McCarthy FP, Saito S, et al. Hypertensive Disorders of Pregnancy: Isshp Classification, Diagnosis, and Management Recommendations for International Practice. *Hypertension* (2018) 72(1):24-43. Epub 2018/06/15. doi: 10.1161/HYPERTENSIONAHA.117.10803.

14. Farzin L, Sajadi F. Comparison of Serum Trace Element Levels in Patients with or without Pre-Eclampsia. *Journal of research in medical sciences : the official journal of Isfahan University of Medical Sciences* (2012) 17(10):938-41. Epub 2013/07/05.

15. Feng JJ, Wang YX. The Measurement and Clinical Significance of Serum Trace Elements in Early - Onset Severe Preeclampsia. *Chinese Journal of Birth Health & Heredity* (2013) 21(05):82-4. doi: 10.13404/j.cnki.cjbhh.2013.05.044.

16. Fenzl V, Flegar-Meštrić Z, Perkov S, Andrišić L, Tatzber F, Žarković N, et al. Trace Elements and Oxidative Stress in Hypertensive Disorders of Pregnancy. *Archives of gynecology and obstetrics* (2013) 287(1):19-24. Epub 2012/08/11. doi: 10.1007/s00404-012-2502-4.

17. Pregnancy NHBPEPWGoHBPi. Report of the National High Blood Pressure Education Program Working Group on High Blood Pressure in Pregnancy. *Am J Obstet Gynecol* (2000) 183(1):S1-S22. Epub 2000/08/02.

18. Gao LY, Wang Y, Wu WW, Feng YL, Yang HL, Wang SP. A Case-Control Study on the Relationship between Maternal Copper and Preeclampsia. *Chin J Dis Control Prev* (2020) 24(12):1447-51. doi: 10.16462/j.cnki.zhjbkz.2020.12.016.

19. Gul AZ, Atakul N, Selek S, Atamer Y, Sarikaya U, Yildiz T, et al. Maternal Serum Levels of Zinc, Copper, and Thiols in Preeclampsia Patients: A Case-Control Study. *Biological trace element research* (2022) 200(2):464-72. doi: 10.1007/s12011-021-02660-y.

20. Acog Practice Bulletin No. 202: Gestational Hypertension and Preeclampsia. *Obstet Gynecol* (2019) 133(1):1. Epub 2018/12/24. doi: 10.1097/AOG.0000000000003018.

21. Guo LL, Guo SL, Li SX, Zhang SY, Li HX, Niu LH, et al. The Study on the Relationship between Trace Elements Content in Whole Blood and Hypertensive Disorder Complicating Pregnancy. *Chinese Journal of Birth Health & Heredity* (2013) (11):4.

22. Ilhan N, Ilhan N, Simsek M. The Changes of Trace Elements, Malondialdehyde Levels and Superoxide Dismutase Activities in Pregnancy with or without Preeclampsia. *Clinical biochemistry* (2002) 35(5):393-7. Epub 2002/09/25. doi: 10.1016/s0009-9120(02)00336-3.

23. Jamal B, Shaikh F, Memon MY. To Determine the Effects of Copper, Zinc and Magnesium in Patients with Pre-Eclampsia. *Journal of the Liaquat University of Medical and Health Sciences* (2017) 16(1):53-7. doi: 10.22442/jlumhs.171610506.

24. Kanagal DV, Rajesh A, Rao K, Shetty H, Shetty PK, Ullal H. Zinc and Copper Levels in Preeclampsia: A Study from Coastal South India. *Int J Reprod Contracept Obstet Gynecol* (2014) 3(2):370-3.

25. Bulletins--Obstetrics ACoP. Acog Practice Bulletin. Diagnosis and Management of Preeclampsia and Eclampsia. Number 33, January 2002. *Obstet Gynecol* (2002) 99(1):159-67. Epub 2005/09/24. doi: 10.1016/s0029-7844(01)01747-1.

26. Keshavarz P, Nobakht MGBF, Mirhafez SR, Nematy M, Azimi-Nezhad M, Afin SA, et al. Alterations in Lipid Profile, Zinc and Copper Levels and Superoxide Dismutase Activities in Normal Pregnancy and Preeclampsia. *The American journal of the medical sciences* (2017) 353(6):552-8. Epub 2017/06/24. doi: 10.1016/j.amjms.2017.03.022.

27. Williams JW, Gary CF, J. LK, L. BS, C. HJ, C. GL, et al. *Williams Obstetrics*. 22ed ed. New York, NY: McGraw-Hill (2005).

28. Lewandowska M, Sajdak S, Marciniak W, Lubiński J. First Trimester Serum Copper or Zinc Levels, and Risk of Pregnancy-Induced Hypertension. *Nutrients* (2019) 11(10). Epub 2019/10/19. doi: 10.3390/nu11102479.

29. Li PZ, Li XY. Trace Elements in Pregnancy-Induced Hypertension and Related Diseases Research. *Guide of China Medicine* (2009) (10):29-31.

30. Lu YH, Han LJ, Zhang L, Ni SN, Tian QY. Correlation between Hypertensive Disorder in Pregnancy and Serum Calcium, Prostaglandin E, Endothelin. *Hebei Medical Journal* (2016) 38(07):1057-9.

31. Maduray K, Moodley J, Soobramoney C, Moodley R, Naicker T. Elemental Analysis of Serum and Hair from Pre-Eclamptic South African Women. *Journal of trace elements in medicine and biology : organ of the Society for Minerals and Trace Elements (GMS)* (2017) 43:180-6. Epub 2017/03/23. doi: 10.1016/j.jtemb.2017.03.004.

32. McKeating DR, Fisher JJ, MacDonald T, Walker S, Tong S, Bennett WW, et al. Circulating Trace Elements for the Prediction of Preeclampsia and Small for Gestational Age Babies. *Metabolomics : Official journal of the Metabolomic Society* (2021) 17(10):90. Epub 2021/09/25. doi: 10.1007/s11306-021-01840-0.

33. Mistry HD, Gill CA, Kurlak LO, Seed PT, Hesketh JE, Méplan C, et al. Association between Maternal Micronutrient Status, Oxidative Stress, and Common Genetic Variants in Antioxidant Enzymes at 15 Weeks׳ Gestation in Nulliparous Women Who Subsequently Develop Preeclampsia. *Free radical biology & medicine* (2015) 78:147-55. Epub 2014/12/03. doi: 10.1016/j.freeradbiomed.2014.10.580.

34. Onyegbule AO, Onah CC, Iheukwumere BC, Udo JN, Atuegbu CC, Nosakhare NO. Serum Copper and Zinc Levels in Preeclamptic Nigerian Women. *Nigerian medical journal : journal of the Nigeria Medical Association* (2016) 57(3):182-4. Epub 2016/07/12. doi: 10.4103/0300-1652.184071.

35. Rafeeinia A, Tabandeh A, Khajeniazi S, Marjani AJ. Serum Copper, Zinc and Lipid Peroxidation in Pregnant Women with Preeclampsia in Gorgan. *The open biochemistry journal* (2014) 8:83-8. Epub 2014/11/18. doi: 10.2174/1874091x01408010083.

36. Ranjkesh F, Jaliseh HK, Abutorabi S. Monitoring the Copper Content of Serum and Urine in Pregnancies Complicated by Preeclampsia. *Biol Trace Elem Res* (2011) 144(1-3):58-62. Epub 2011/04/14. doi: 10.1007/s12011-011-9026-9.

37. Cunningham FG, Whitridge WJ. *Williams Obstetrics*. 23ed ed. New York, NY: McGraw-Hill (2010).

38. Rathore S, Gupta A, Batra HS, Rathore R. Comparative Study of Trace Elements and Serum Ceruloplasmin Level in Normal and Pre-Eclamptic Pregnancies with Their Cord Blood. *Biomedical Research-India* (2011) 22(2):207-10.

39. Sak S, Barut M, Çelik H, Incebiyik A, Ağaçayak E, Uyanikoglu H, et al. Copper and Ceruloplasmin Levels Are Closely Related to the Severity of Preeclampsia. *The journal of maternal-fetal & neonatal medicine : the official journal of the European Association of Perinatal Medicine, the Federation of Asia and Oceania Perinatal Societies, the International Society of Perinatal Obstet* (2020) 33(1):96-102. Epub 2018/06/12. doi: 10.1080/14767058.2018.1487934.

40. Samar A, Wang DL, Iskandar X. Correlation between Gestational Hypertension and Serum Vitamin D, Trace Element Level *Journal of Xinjiang Medical University* (2020) 43(5):597-600,6. doi: 10.3969/j.issn.1009-5551.2020.05.015.

41. Sarwar MS, Ahmed S, Ullah MS, Kabir H, Rahman GK, Hasnat A, et al. Comparative Study of Serum Zinc, Copper, Manganese, and Iron in Preeclamptic Pregnant Women. *Biol Trace Elem Res* (2013) 154(1):14-20. Epub 2013/06/12. doi: 10.1007/s12011-013-9721-9.

42. Serdar Z, Gür E, Develioğlu O. Serum Iron and Copper Status and Oxidative Stress in Severe and Mild Preeclampsia. *Cell biochemistry and function* (2006) 24(3):209-15. Epub 2005/05/18. doi: 10.1002/cbf.1235.

43. Ugwuja EI, Ejikeme BN, Ugwu NC, Obeka NC, Akubugwo EI, Obidoa O. Comparison of Plasma Copper, Iron and Zinc Levels in Hypertensive and Non-Hypertensive Pregnant Women in Abakaliki, South Eastern Nigeria. *Pak J Nutr* (2010) 9(12):1136-40.

44. Yang LT, Gu LP, Zhang WY, Fan LM. The Study on the Relationship between Hypertensive Disorder Complicating Pregnancy and the Serum Zinc, Copper, Iron, Maganese. *Maternal and Child Health Care of China* (2007) (29):4082-5.

45. Le J. *Obstetrics and Gynecology*. 6th ed. Beijing, China: People's Medical Publishing House (2004) 2004.
